# Supplementary material for: Ultrafast perturbation maps as a quantitative tool for testing of multi-port photonic devices
Source: Nat Commun. 2018 Jun 8;9:2246. doi: 10.1038/s41467-018-04662-2 (PMC5993788; doi:10.1038/s41467-018-04662-2)
Supplement: Supplementary file 1 — Supplementary Information [file 41467_2018_4662_MOESM1_ESM.pdf]

Supplementary Information for "Ultrafast perturbation maps as a quantitative tool for testing of multi-port photonic devices"

Kevin Vynck,[1,*] Nicholas J. Dinsdale,[2,3] Bigeng Chen,[2] Roman Bruck,[2] Ali Z. Khokhar,[3] Scott A. Reynolds,[3] Lee Crudgington,[3] David J. Thomson,[3] Graham T. Reed,[3] Philippe Lalanne,[1] and Otto L. Muskens[2,†]

[1]*LP2N, CNRS - Institut d'Optique Graduate School - Univ. Bordeaux, F-33400 Talence, France*

[2]*Physics and Astronomy, Faculty of Physical Sciences and Engineering, University of Southampton, Southampton, UK*

[3]*Optoelectronics Research Centre, University of Southampton, Southampton, UK*

[*] kevin.vynck@institutoptique.fr

[†] o.muskens@soton.ac.uk

Supplementary Note 1. Importance of local-field corrections

As discussed in the main text of this work, the coupling coefficient C_{mn} in Eq. (6) of the main text is defined from the total fields in the perturbed system and not from the background fields. To be able to predict C_{mn} accurately for realistic perturbations from only two computations, the former should thus be expressed as a function of the latter.

In Supplementary Figure 1, we show that neglecting the difference between total and background fields impedes the recovery of highly-accurate results for the small cylindrical perturbations considered in the main text ($2R = 10$ nm, $\Delta n = -0.25$). While the general trend is correct, approximating $\mathbf{E} \approx \mathbf{E}_\mathrm{b}$ results in a net difference in amplitude. A similar effect is observed for larger perturbations. Indeed, neglecting the dephasing term in Eqs. (11) or (12) of the main text and applying Eq. (2) of the main text to obtain $\Delta T/T$ will essentially result in a wrong amplitude.

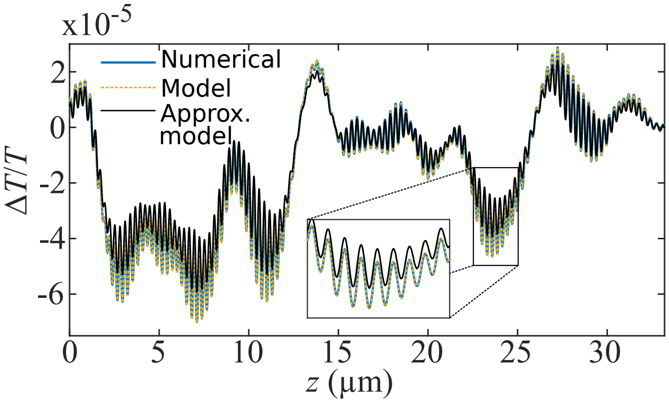

Supplementary Figure 1: Impact of local-field correction on accuracy of the model. Same as Fig. 2c, with an additional curve showing $\Delta T/T$ predicted by replacing Eq. (7) of the main text by $\mathbf{E}(\mathbf{r}_0) = \mathbf{E}_\mathrm{b}(\mathbf{r}_0)$ (black solid curve). It appears clearly that the local-field correction is necessary to obtain highly-accurate results.

Supplementary Note 2. Remaining perturbation maps for 1×4 and 3×3 MMIs

Supplementary Figures 2 and 3 show the port combinations for the 1×4 and 3×3 MMI devices, respectively, that bare some symmetry to the ones shown in the main text.

In the case of the 1×4 device, shown in Supplementary Figure 2, agreement is outstanding once again, with the second-to-bottom coupling resulting in even better correlation than the second-to-top. This difference primarily stems from the larger region of increased $\Delta T/T$ found towards to the top of the device for the second-to-top output. The correlation plots show the device is less sensitive to width variations than the 3×3 MMI, due to the W step size representing a smaller fraction of the device's width. The 2% correlation contour continues to predict a ~ 0.1 µm wider device.

The 3×3 device has the most symmetry of the MMIs investigated here. The five remaining maps shown in Supplementary Figure 3 confirm the width reduction by ~ 0.1 µm.

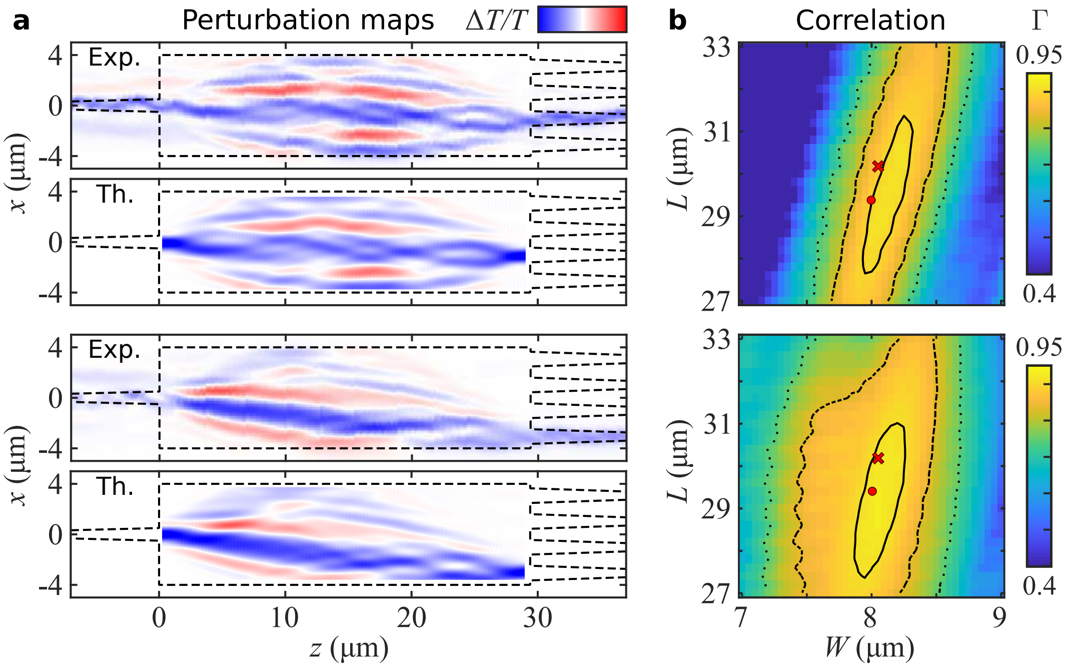

Supplementary Figure 2: Remaining perturbation maps and correlation Γ on the 1×4 MMI. a. Experimental and theoretical perturbation maps for the transmittance between the input waveguide fundamental mode and the bottom two waveguides fundamental modes. The amplitude of the colour bar is ± 0.25 for the experimental maps and ± 0.35 for the theoretical ones. **b.** 2D correlation maps for varying theoretical MMI region dimensions L and W, for the two considered output modes. The red dot and cross indicate the MMI design and SEM measured dimensions respectively, and the contour lines indicate a 2% (solid), 10% (dash) and 20% (dot) decrease of the correlation with respect to the maximum correlation.

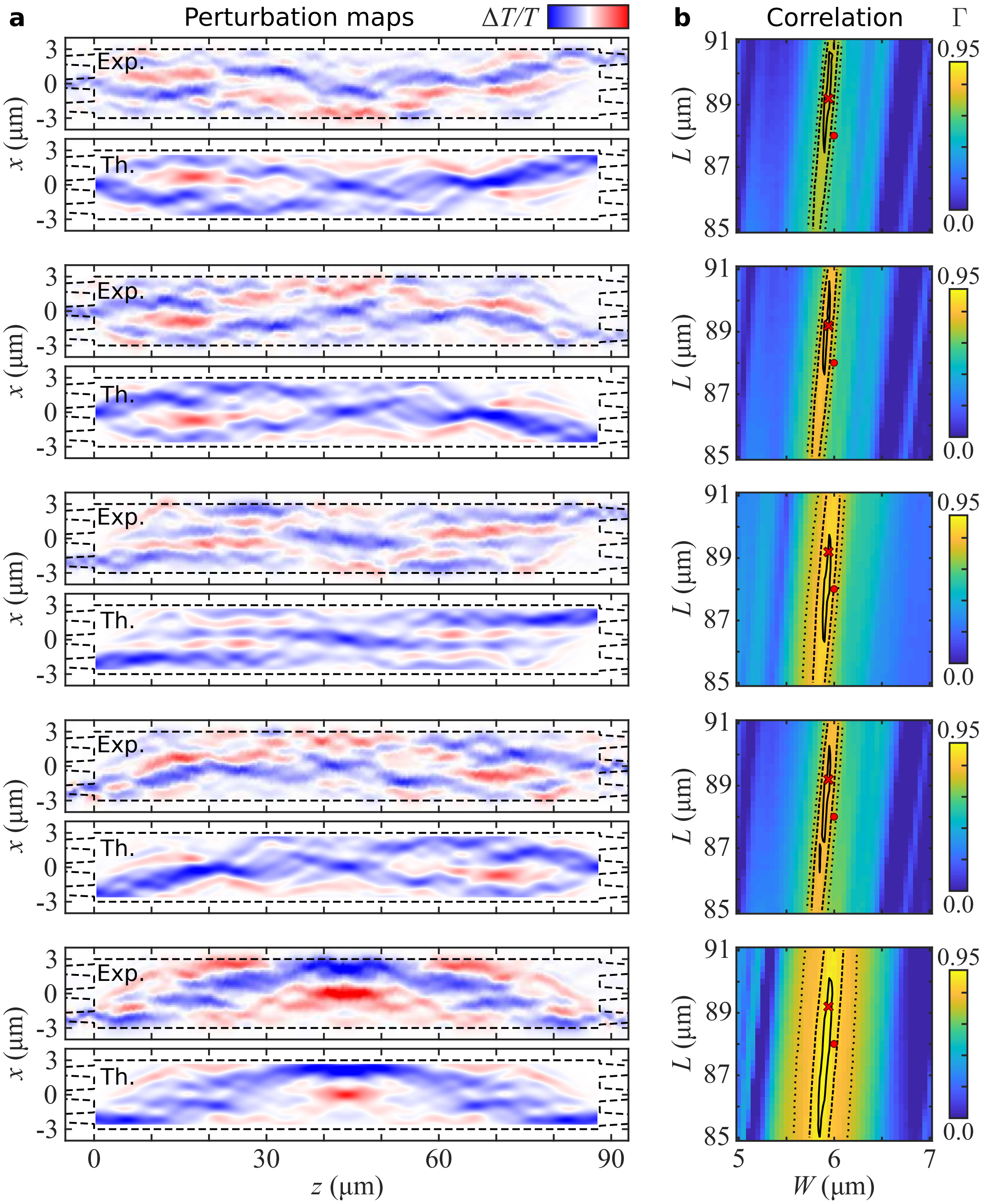

Supplementary Figure 3: Remaining perturbation maps and correlation Γ on the 3×3 MMI. a. Experimental and theoretical perturbation maps for the transmittance for the fundamental mode coupling between remaining ports. The amplitude of the colour bar is ±0.3 for the experimental maps and ±0.4 for the theoretical ones. The scale in the propagation axis has been halved. **b.** 2D correlation maps for varying theoretical MMI region dimensions L and W.

Supplementary Note 3. Original experimental results on 1×2 MMI

In Supplementary Figure 4, we report the UPMS results on a 1×2 MMI with a design length $L = 33.2$ µm and width $W = 6.0$ µm. Like the 1×4 and 3×3 MMIs, this device is based on rib waveguides and is not covered by silica. Very good agreement between the experimental and theoretical perturbation maps is observed. The 2D correlation maps show a maximum in correlation of 0.8610 and 0.9135 for the top and bottom outputs, respectively. The difference in these values suggests some asymmetry in the fabricated device. Close inspection of the perturbation maps from top and bottom outputs shows some differences, particularly toward the beginning of the MMI input. For the top output, one also observes a second local maximum, however the largest correlation coefficients occur around the correct dimensions.

According to Eq. (6) of the main text, perturbation maps are symmetric upon reversing the direction of the light flow through the device. Indeed, identical experimental maps are obtained when light is launched into one of the outputs and collected from the input port as shown experimentally in Supplementary Figure 5, obtained by rotating the device by 180°. We can identify the same deviations between the two output ports in the reversed maps, unequivocally showing that they are related to structural variations of the device and not to an experimental artifact of the imaging system. Interestingly, the same asymmetry can be seen around the single port side as in Supplementary Figure 4.

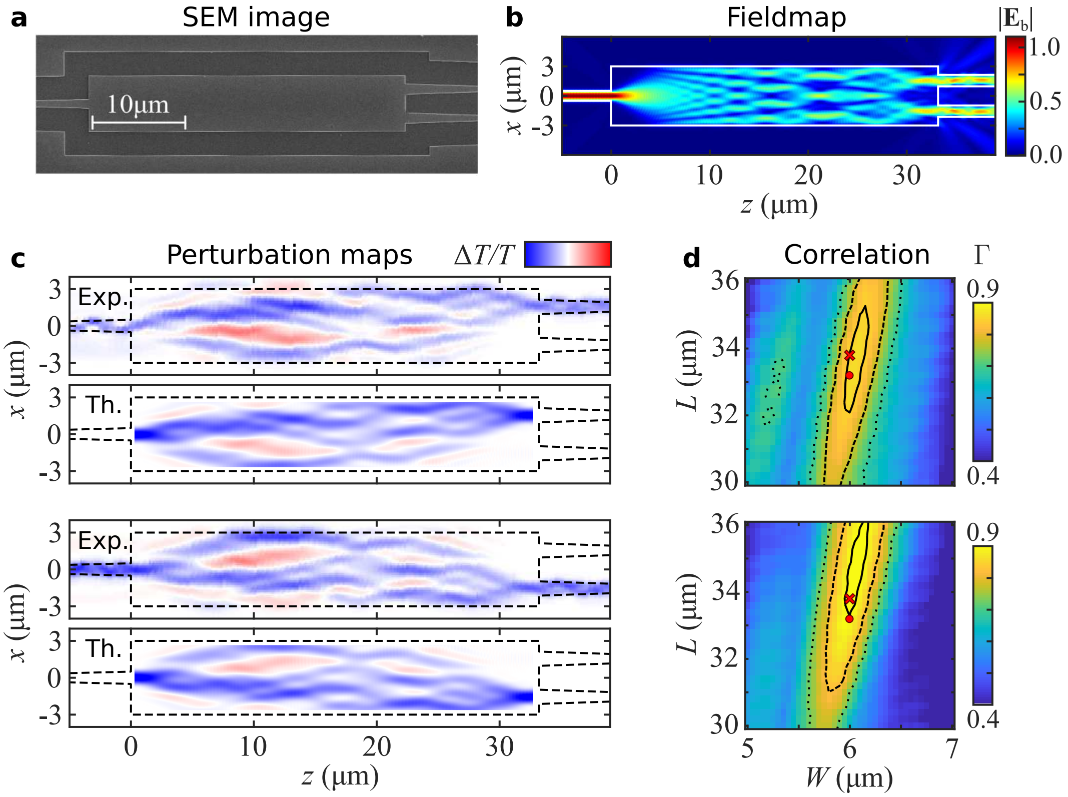

Supplementary Figure 4: Experimental investigation of a 1×2 MMI and comparison with predictions. a. SEM image of the MMI with design length $L = 33.2$ µm and width $W = 6.0$ µm, and fabricated length $L = 33.8$ µm and width $W = 6.0$ µm. **b.** Calculated fieldmap of the device for excitation by the fundamental mode of the input waveguide. **c.** Experimental and theoretical perturbation maps for the transmittance between the input waveguide fundamental mode and the top and bottom waveguides fundamental modes, showing a remarkable agreement in their details. The amplitude of the colour bar is ±0.25 for the experimental maps and ±0.35 for the theoretical ones. **d.** 2D correlation maps for varying theoretical MMI region dimensions L and W, for the two considered output modes. The red dot and cross indicate the MMI design and SEM measured dimensions, respectively, and the contour lines indicate a 2% (solid), 10% (dash) and 20% (dot) decrease of the correlation with respect to the maximum correlation.

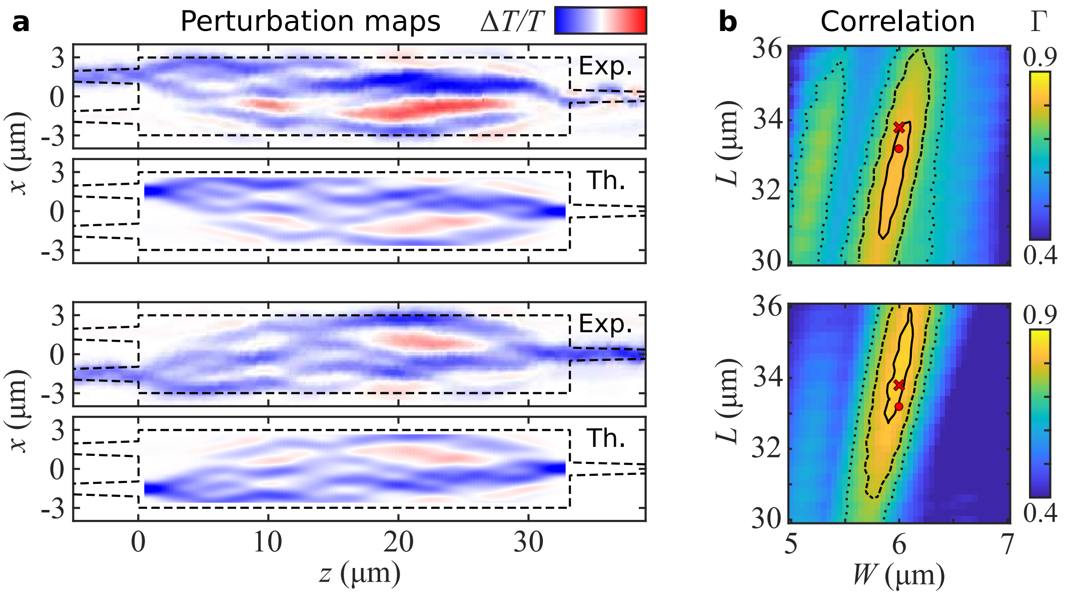

Supplementary Figure 5: Experimental verification of reciprocity on a 1×2 MMI. Same as in Supplementary Figure 4c-d, but with the chip rotated by 180° such that light is incident from one of the two ports. The amplitude of the colour bar is ± 0.25 for the experimental maps and ± 0.35 for the theoretical ones. The same device asymmetry is seen between the experimental perturbation maps and correlation plots as in the forward propagation device.

Supplementary Note 4. Comparison of predicted perturbation maps using various MMI parameters

As shown in the main text, the correlation between the measured perturbation map and a set of predicted perturbation maps for various MMI dimensions allows the detection of potential deviations in structural parameters from design. In the case of the 3×3 MMI, the correlation indicates that the maximum correlation is obtained for a MMI width reduction by about 0.1 µm. In the main text, we show the predicted perturbation maps for the design parameters. Here, we compare the experimental perturbation map to predicted perturbation maps with various MMI parameters.

Supplemetary Figure 6 shows the result for the coupling between the fundamental modes of the top input and top output waveguides, for which the correlation is the highest with the MMI design parameters for this set of measurements (87.32 %). Using the MMI dimensions retrieved from SEM, the agreement improves (93.22 %), especially in the central region. The perturbation maps yielding similar correlation (first and second maxima) are almost identical by eye.

Supplementary Figure 7 shows the result for the coupling between the fundamental modes of the middle input and bottom output waveguides, which is one of the poorer correlations with MMI design parameters for this set of measurements (64.62 %). Using the MMI dimensions retrieved from SEM, the correlation improves quite significantly (80.61 %), with a visual agreement that is readily better. Taking the dimensions yielding the first and second correlation maxima does not significantly improve the agreement.

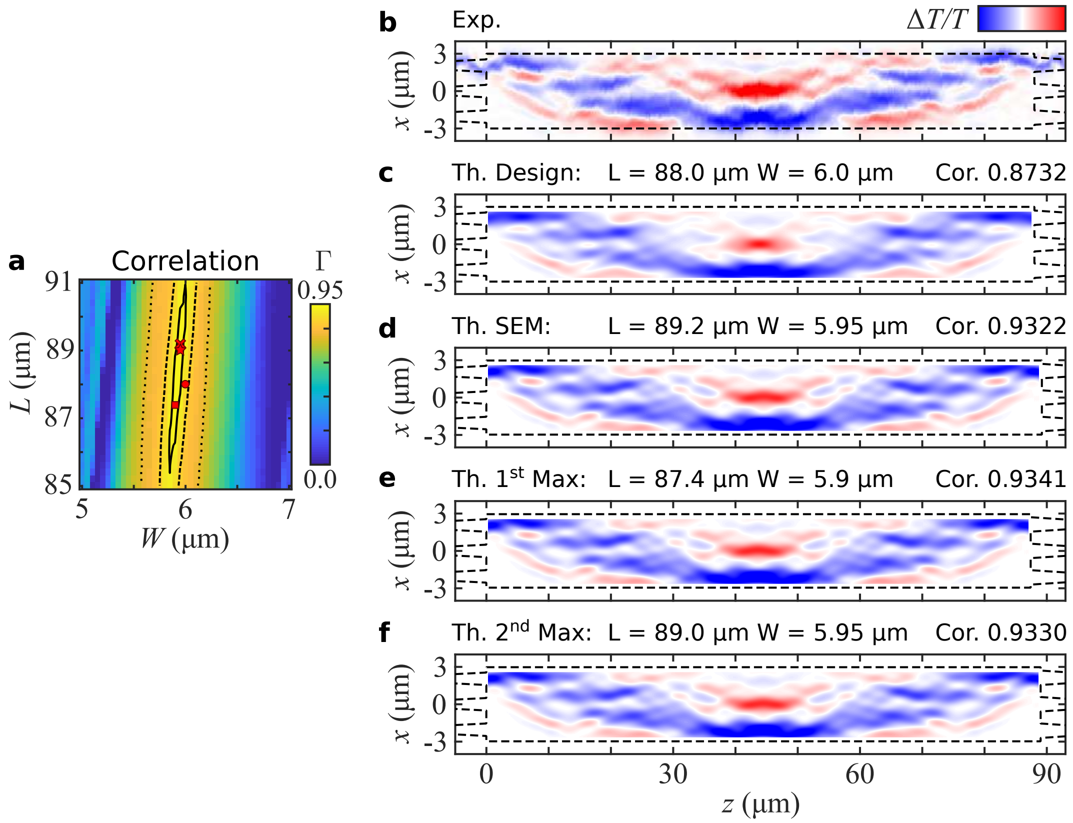

Supplementary Figure 6: Comparison of predicted perturbation maps using design, SEM and maximum correlation device parameters for the best correlating port coupling. a. 2D correlation map for the top-to-top coupling of the 3×3 MMI device, where markers indicate the design (circle), SEM (cross) and first (square) and second (star) maximum correlation dimensions. **b-f.** Perturbation maps for the experimental and modeled design, SEM and first and second maximum correlation device dimensions. The amplitude of the colour bar is ± 0.3 for the experimental maps and ± 0.4 for the theoretical maps.

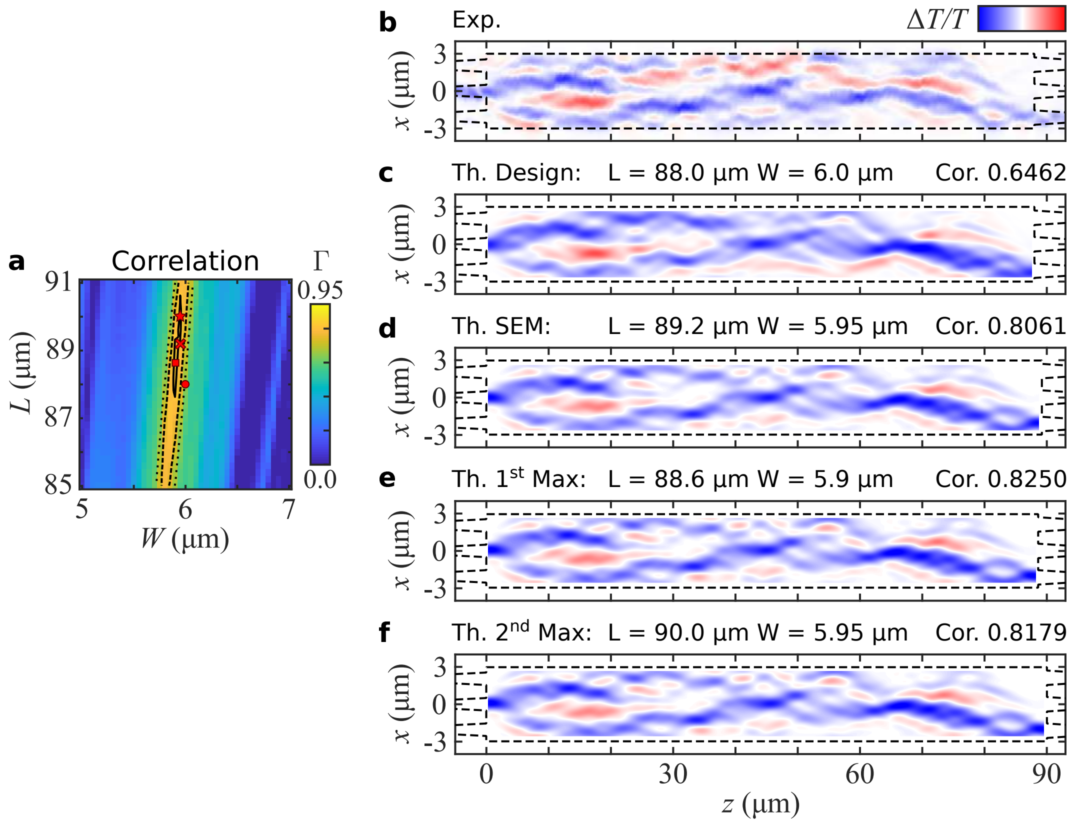

Supplementary Figure 7: Comparison of predicted perturbation maps using design, SEM and maximum correlation device parameters for one of the poorer correlating port coupling. a. 2D correlation map for the middle-to-bottom coupling of the 3×3 MMI device, where markers indicate the design (circle), SEM (cross) and first (square) and second (star) maximum correlation dimensions. **b-f.** Perturbation maps for the experimental and modeled design, SEM and first and second maximum correlation device dimensions. The amplitude of the colour bar is ± 0.3 for the experimental maps and ± 0.4 for the theoretical maps.

Supplementary Note 5. Field intensity recovery from perturbation maps

Here, we will show that the field intensity propagating in a photonic device can be recovered from perturbation map measurements under certain circumstances. Starting from Eq. (2) of the main text,

$$\frac{\Delta T}{T} = \frac{|C_{mn}|^2}{|t^0_{mn}|^2} + 2\mathrm{Re}\left[\frac{C_{mn}}{t^0_{mn}}\right], \tag{1}$$

we make a first assumption, which is that the permittivity variation acts as a small perturbation on the transmission, i.e. $|C_{mn}| \ll |t^0_{mn}|$. To first order in C_{mn}/t^0_{mn}, we are therefore left with

$$\frac{\Delta T}{T} \approx 2\mathrm{Re}\left[\frac{C_{mn}}{t^0_{mn}}\right]. \tag{2}$$

We proceed by inserting an analytical expression of the coupling coefficient in Eq. (2). For the sake of the example, we take Eq. (12) of the main text, which applies to large low-index contrast perturbations, leading to

$$\frac{\Delta T}{T} \approx 2\mathrm{Re}\left[\frac{1}{t^0_{mn}}\frac{i\omega\epsilon_0}{4}\exp\left[i\frac{\omega}{c}\Delta n d\right]\Delta\epsilon\int_{S_\mathrm{p}}\mathbf{E}^+_{\mathrm{b},m}(\mathbf{r})\cdot\mathbf{E}^+_{\mathrm{b},n}(\mathbf{r})d\mathbf{r}\right]. \tag{3}$$

At this point, we make a second assumption, which is that the transmission to the output mode $|t^0_{mn}|$ is nearly unity. In this limit, Lorentz reciprocity indicates that the ingoing propagating field generated from the output mode should be the complex conjugate of the propagating field generated from the input mode, i.e. $\mathbf{E}^+_{\mathrm{b},n}(\mathbf{r}) = \left[\mathbf{E}^+_{\mathrm{b},m}(\mathbf{r})\right]^\star$. Equation (3) finally becomes

$$\frac{\Delta T}{T} \approx \frac{\omega\epsilon_0}{2}\mathrm{Re}\left[\frac{i\exp\left[i\omega\Delta n d/c\right]\Delta\epsilon}{\exp\left[i\phi_t\right]}\right]\int_{S_\mathrm{p}}|\mathbf{E}^+_{\mathrm{b},m}(\mathbf{r})|^2 d\mathbf{r}, \tag{4}$$

where $\phi_t = \arg(t^0_{mn})$. The term Re [...] being bounded on $\mathbb{R}$, we conclude that, to first order in C_{mn}/t^0_{mn} and for near unitary transmission, the perturbation map $\frac{\Delta T}{T}$ should be directly proportional to the field intensity integrated over the perturbation surface area S_p.
